# Evaluating a computerized maintenance management system in a low resource setting

**DOI:** 10.1007/s12553-021-00524-y

**Published:** 2021-02-27

**Authors:** Farah Beniacoub, Fabrice Ntwari, Jean-Paul Niyonkuru, Marc Nyssen, Stefaan Van Bastelaere

**Affiliations:** 1 Enabel, Belgian development agency, Rue Haute 147, 1000 Brussels, Belgium; 2 Central Directorate for Health Infrastructure and Equipment Management of the Ministry of Health (MoH), Avenue Pierre Ngendandumwe, Bujumbura, Burundi; 3 Department of Public Health, Vrije Universiteit Brussel (VUB), Laarbeeklaan 103, 1090 Brussels, Belgium

**Keywords:** Maintenance, Management information systems, Ancillary information systems, Biomedical technology, HTMS

## Abstract

This study documents the setup and roll-out of a Computerized Maintenance Management System (CMMS) in Burundi’s resource constrained health care system between 1/04/2017 and 31/03/2020. First, in 2017 a biomedical assets ontology was created, tailored to the local health system and progressively mapped on international GMDN (Global Medical Devices Nomenclature) and ICMD (International Classification and Nomenclature of Medical Devices) classifications. This ontology was the cornerstone of a web-based CMMS, deployed in the Kirundo and Muramvya provinces (6 health districts, 4 hospitals and 73 health centers).

During the study period, the total number of biomedical maintenance interventions increased from 4 to 350 per month, average corrective maintenance delays were reduced from 106 to 26 days and the proportion of functional medical assets grew from 88 to 91%.

This study proves that a sustainable implementation of a CMMS is feasible and highly useful in low resource settings, if (i) the implementation is done in a conducive technical environment with correct workshops and maintenance equipment, (ii) the active cooperation of the administrative authorities is ensured, (iii) sufficient training efforts are made, (iv) necessary hardware and internet connectivity is available and (v) adequate local technical support can be provided.

## Introduction

Poor management of biomedical equipment and health care infrastructure is a problem of most health systems in low income countries. The most obvious reasons are:

**Lack of accurate information:** Very few Ministries of Health (MoH) have an accurate asset inventory at national, sub-national or health facility levels (diagnostic equipment, medical rolling stock, buildings, grounds or IT equipment [1, 2]). It is even more difficult to obtain up-to-date information about the functional status, the maintenance history and the maintenance planning of equipment and buildings. This lack of essential information makes adequate planning, management and monitoring of major public investments in health care equipment and infrastructure very difficult[1]: numerous buildings are in dilapidated condition due to lack of maintenance, scrap yards are plenty of defective but in some cases perfectly repairable medical devices, biomedical equipment is distributed in an irrational way and sometimes cannot be put into operation due to simple lack of electricity or technical knowledge required for installation and configuration.

**Absence of adapted, standardized nomenclature:** The absence of national or international standardized nomenclatures for the identification of biomedical equipment and health system infrastructure has been observed in multiple low resource countries [2, 3]. Often user-generated descriptions in free text are in use, resulting in typing errors and non-standard acronyms. The absence of an unambiguous ontology for biomedical engineering makes the exploitation of the available biomedical inventory time-consuming and error-prone. Nor does it allow health administrations to automatically evaluate the technical platform and infrastructure availability and maintenance status to national standards, insofar as these have been defined.

**Shortage of qualified and skilled biomedical technicians:** A marked shortage of skilled biomedical technicians in sub-Saharan Africa has been currently observed. This is particularly evident in rural areas and for the public sector. It contributes to the phenomenon of expensive maintenance contracts offered by international manufacturers, relying on expensive international technical personnel, which often proves impossible due to budgetary restrictions.

**Lack of appropriate maintenance equipment, workshops and assistance:** The scarce local technicians often do lack the appropriate equipment to carry out common repairs. They work in isolated settings without technical supervision or assistance. Therefore, necessary preventive maintenance tasks and curative repairs are not performed correctly or in due time, resulting in a very short functional lifetime of the biomedical equipment and consequently the frequent and long-term unavailability of sometimes essential diagnostic and/or therapeutic services for the patients [4, 5].

In the past decade, digitalization was introduced in the health care systems of many developing countries. National e-health strategies were developed (e.g. Burundi’s *Plan National de Développement de l'Informatique de Santé—PNDIS*) in which priority was given to internet connectivity, the setting up of national data warehouses including geo-referencing of aggregated health data, the computerization of hospitals and health centers or the automation of the pharmaceutical supply chain. Digitalization of biomedical equipment and health infrastructure management was unfortunately given a lower priority in these plans and it was seldom integrated in the national health management information systems. Moreover, countries are facing important challenges when implementing these e-health strategies, such as (i) unavailability of a central e-health authority which can coordinate the many over-priced donor-driven e-health projects, (ii) low-bandwidth or not widely available internet connectivity, (iii) lack of digital literacy (iv) lack of guidance on standards.

### Research question

Our hypothesis is that implementation of a Computerized Maintenance Management System (CMMS) contributes to a more effective and efficient management of biomedical equipment and health infrastructure in a low resource setting.

## Materials and methods

The study population of our action research included the Central Directorate for Health Infrastructure and Equipment Management of the MoH. At decentral level it included all public hospitals (n = 4) and health centers (n = 73) of the provinces of Muramvya and Kirundo.

The action research consisted of the following elements:

1. Developing a standardized nomenclature for all biomedical equipment and health infrastructure.
2. Establishing unambiguous quantitative standards for biomedical equipment and infrastructure based on the health norms of the MoH.
3. Realizing a digital inventory of all biomedical material and health infrastructure based on the developed nomenclature.
4. The development of a digital information system for the management of inventories, maintenance plans and maintenance activities and making the system available to the central services of the MoH and to the decentral level, i.e. the maintenance technicians in the 2 provinces concerned.
5. The setup of biomedical workshops in the 2 concerned provinces with maintenance equipment and skilled technicians according to the national norms.
6. The training of technical personnel of the central services of the MoH and of all available maintenance technicians in the 2 provinces.

The action research was analyzed in a quantitative and qualitative way covering (i) the use of the CMMS over a period of 3 years (2017–2020), (ii) the study of the feasibility, results and sustainability of the intervention and (iii) identification of any relevant failure- and success factors to be taken into account for future CMMS implementations in low resource settings.

## Results

In an initial phase, the ontology was developed for both biomedical equipment and medical infrastructure assets as described below:

### Biomedical assets nomenclature

For biomedical equipment categories, local terminologies familiar to local maintenance technicians were used as a starting point. This resulting local nomenclature was progressively mapped to the international standards like GMDN (in 2018) [6] and ICMD-11 (in 2020) [7]. On a total of 131 local nomenclature codes, a corresponding GMDN code could be found for 97 (74%) items and a matching ICMD code for 83 (63%) items.

Information in the form of a 5-digit numeric GMDN Code is cross-referenced to a precisely defined Term Name and Definition, as seen in this example:

GMDN Term Name: Scalpel, single-use.

GMDN Code: 47,569

GMDN Definition: “A sterile, hand-held, manual surgical instrument constructed as a one-piece handle and scalpel blade (not an exchangeable component) used by the operator to manually cut or dissect tissue. The blade is typically made of high-grade stainless steel alloy or carbon steel and the handle is often made of plastic. This is a single-use device”.

Disadvantages of the GMDN classification were the fact that it was not available for free, that the code mapping took significantly longer to complete due to its much higher granularity compared to the ICMD classification and that GMDN matching also generated three times more ambiguous mappings, ultimately requiring an expert to make a choice between different possible candidate codes.

### Infrastructure ontology

No useful international coding system could be found for classification of infrastructure assets. To this end, a new tri-axial nomenclature was locally developed, which took into account (i) the location of an item in the health pyramid (district hospital, health center …), (ii) the functional belonging of an item (radiology, laboratory, administration, ancillary building …) and (iii) its technical classification (roof truss, floor, network cabling …). The resulting infrastructure nomenclature contained 102 codes for the combinations of the first 2 axes each of which could be combined with 63 technical classification codes.

### Health facility asset norms

Subsequently, existing Burundian quantitative national norms for infrastructure and biomedical equipment in district hospitals and health centers (numbers of operating theaters, reanimation sets, hospital beds, ECG machines etc.) were updated using the developed ontology for biomedical assets. The objective was to obtain an official reference against which the equipment status of each public health facility can be unambiguously assessed in order to enable better future investment planning and a more efficient organization of maintenance activities.

### Baseline inventory

Before the start of the study, data on the biomedical assets of the public health facilities in Burundi had already been collected using an Akvo application on Android smartphones, with which technical characteristics, photos and descriptions of biomedical equipment and infrastructure were recorded according to WHO guidelines [1] in a semi-structured manner for each of the studied health facilities. This inventory was recuperated and migrated into the new system. Using the previously developed biomedical assets ontology, this baseline inventory could be initiated before even starting the actual development of the CMMS. This resulted in an initial list of 647 infrastructure items (only taking into account the first two coding axes) and 745 equipment items for both provinces.

### CMMS functionalities

Before the start of the study, about 20 hospitals in Burundi already had a digital hospital information system with an integrated but seldom used CMMS module. In order to avoid unnecessary introduction of new information systems and to remain interoperable with the information systems already used by these hospitals, it was decided to further build on their public Java programming libraries for the development of the CMMS covering not only the hospitals but the whole district (including district offices and health centers).

The following functionalities, tailored to the specific needs of the Burundian health system, were developed:

1. Management of equipment and infrastructure with the local ontologies and with international biomedical asset nomenclatures.
2. Health facility management according to the hierarchical organizational structure of the Burundian health care system, with differentiated access rights to different categories of users per health facility (hospitals and health centers) and levels (national, provincial, district).
3. Inventory of biomedical equipment and medical infrastructure, based on the previously developed ontology, including administrative, financial and technical characteristics of each asset and the storage of photos and any relevant asset documentation (manuals, loan agreements, inspection reports …).
4. The planning of preventive maintenance and the management of the requests for corrective interventions in case of equipment breakdowns. For that purpose, standardized maintenance schemes have been developed for 68 (52%) equipment and 29 (28%) infrastructure codes.
5. The registration of all maintenance activities including the identity of responsible maintenance technician, the outcome and the costs of the intervention.
6. Automatic generation of reports and dashboards relating to the inventory status, maintenance progress and the extent to which the national quantitative standards for biomedical infrastructure and equipment are complied with.

### CMMS implementation

The CMMS was developed as a central internet-accessible server application with a secure web interface. The application was developed in French as an open source Java project with a MySQL database backend and running on an Apache Tomcat server. The choice for this architecture was partly determined by the fact that (i) Burundi has a fairly broad coverage of 3G / 4G internet access, (ii) the registered data is automatically and permanently available in a central location, (iii) the hardware infrastructure costs are kept to a minimum and (iv) updates and maintenance only need to be performed in one single place. In order to also enable the registration of biomedical asset data (including photos) in remote health centers where internet connectivity is not available, an offline registration module was developed, enabling maintenance technicians to enter data on stand-alone devices in off-line mode for later synchronization with the central server.

### Training

Most maintenance technicians entered in unknown territory with the new CMMS application. Except for some basic knowledge of using Excel and Word, none of them had prior knowledge about a CMMS application. The average IT knowledge among the maintenance technicians was rather low. Therefore, a train-the-trainer approach was chosen, in which, in addition to the end users, expert users from the MoH received extensive training with regard to advanced functionalities and CMMS system management. End users have been successively trained multiple times on a steadily growing complexity of the software [8]. A team of 3 local private computer engineers in Bujumbura was additionally trained to be able to respond on demand (through a maintenance contract) in case of technical failures that can’t be resolved by the MoH’s own expert users.

Although the technical learning curve for using the application was quite short (1 week of training for the maintenance technicians), the need for additional training in biomedical maintenance procedures and strategies developed by the MoH became obvious very soon. For example, many maintenance technicians were relatively new to the sector, and others had developed their own working methods over time which were not always consistent with the procedures implemented in the CMMS. Therefore, the roll-out of the CMMS application got off to a relatively slow start. But the maintenance technicians have been monitored closely and over time they gradually discovered the benefits that could be derived from the application when performing their daily tasks. Repeated additional training was tailored to the needs they expressed and therefore fairly well attended which eventually resulted in a steadily increasing use of the CMMS.

### System usage

**Inventory:** the initial baseline inventory with 1392 registered biomedical assets has been progressively expanded to 2906 permanently updated asset records after 3 years of operation. In the same period, 70 defective equipment items have been disposed of.

**Fig. 1:**
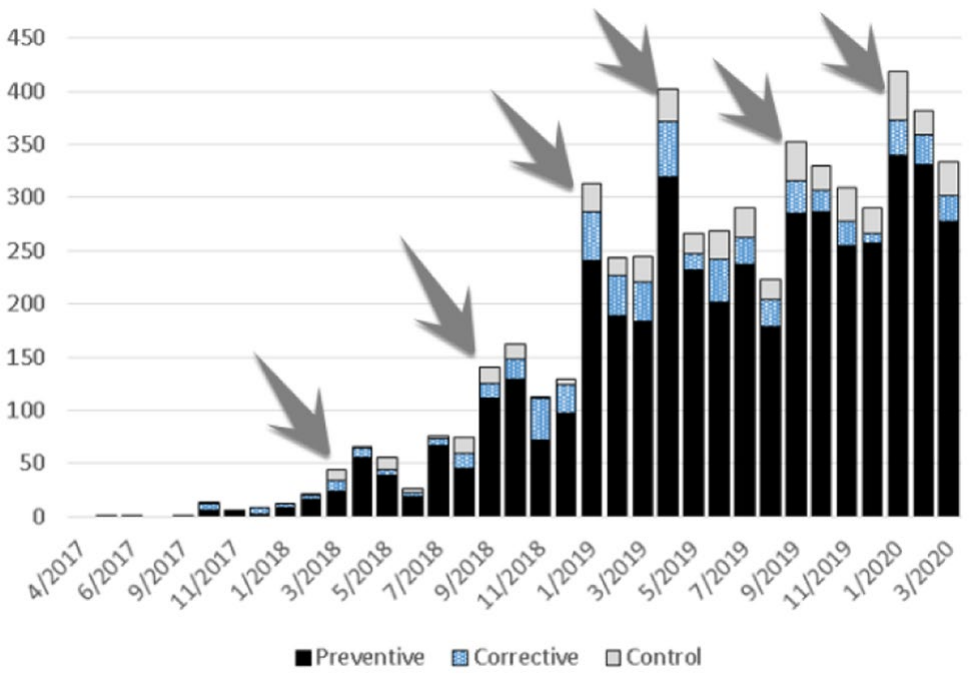
Monthly number of maintenance interventions

**Total number of interventions:** Fig. 1 shows that the monthly number of maintenance interventions on these assets has also seen exponential growth from 4 in the first half of 2017 (baseline), to 20 early 2018, 230 early 2019 and around 350 in March 2020; the arrows indicate the important role periodically repeated training efforts have played in this. On an average basis, preventive maintenance has been responsible for the bulk of the growth in maintenance activity by taking 89% of the total interventions.

### Qualitative and quantitative analysis of the interventions

**Fig. 2:**
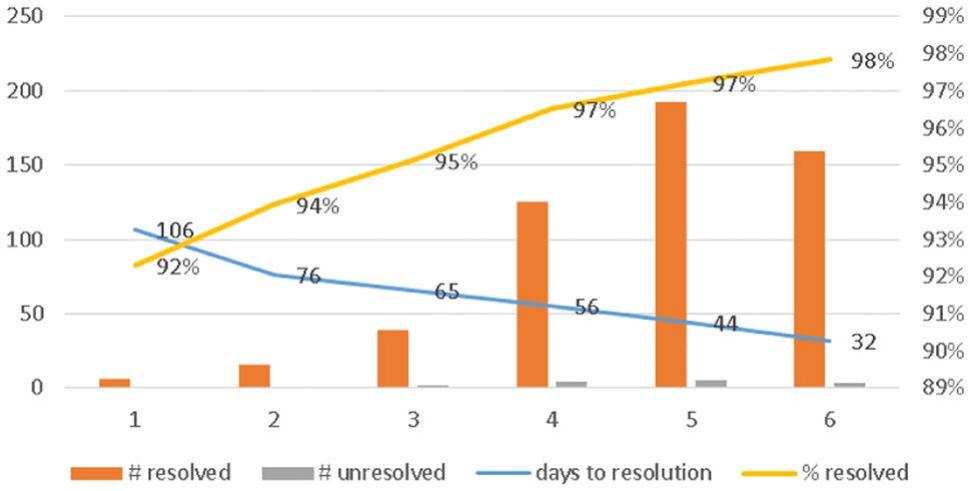
Quality of corrective maintenance interventions

The analysis of the maintenance tasks demonstrated a favorable effect on the quality of the service that was provided by maintenance technicians in the 2 studied provinces. Firstly, the average response time to requests for corrective maintenance in case of biomedical equipment breakdowns, measured between the day of the application for assistance and the first intervention, decreased from 106 days in April 2017 to 26 days in March 2020 (Fig. 2). In the same period, the time that was needed to solve a maintenance problem dropped from 106 to 32 days.

Secondly, whilst the corrective maintenance workload grew from 6 interventions in the first semester to 159 interventions in the sixth semester of the study, simultaneously the proportion of successfully solved cases improved from 92 to 98% (Fig. 2).

From April 2017 to march 2020, on a total of 635 interventions for corrective maintenance, 56% of the requests were issued by district hospitals, 38% by health centers and 6% by administrative structures. 61% of the interventions were related to infrastructure assets and 39% to equipment. During the last 18 months of the study, increasing numbers of preventive maintenance interventions were accompanied by a declining trend in (more expensive) corrective interventions, both in district hospitals and health centers, but this correlation was not statistically significant.

### Functional capacity ratio F

In order to determine whether this growing number of maintenance interventions had an impact on the operational status of the biomedical heritage of the health facilities, the *functional capacity ratio F* was evaluated every six months, reflecting the proportion of active assets in functional condition:

$${\text{F = }}\frac{{{\text{A}}_{{{\text{tf}}}} {\text{ - A}}_{{{\text{df}}}} }}{{{\text{A}}_{{\text{t}}} {\text{ - A}}_{{\text{d}}} }} \times 100$$

where

F = functional capacity ratio in %

A_tf_ = total number of functional assets registered in

the inventory.

A_df_ = total number of functional assets in the inventory

that were decommissioned.

A_t_ = total number of assets registered in the inventory

A_d_ = total number of decommissioned assets

registered in the inventory.

**Fig. 3:**
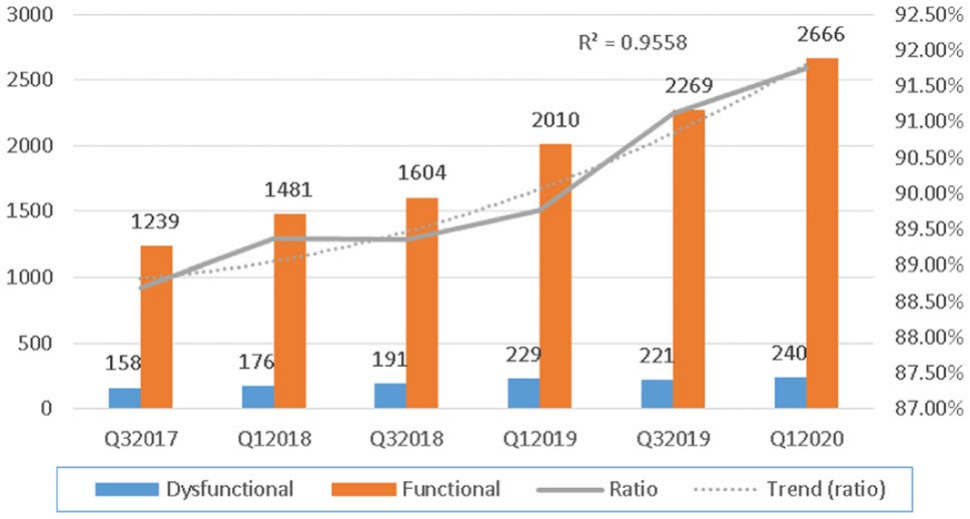
Evolution of functional vs dysfunctional assets

The results, as shown in Fig. 3, teach us that a rather modest but statistically significant improvement in F was observed growing from 88.69% in 2017 to 91.74% in 2020 (polynomial regression second order R = 0.9776). Interestingly, the obtained F-values correlate strongly with the absolute number of preventive maintenance interventions performed (linear regression R = 0.9524 and *p* < 0.005). (Table 1)

## Discussion

During our study period from April 2017 till March 2020 we successfully implemented a CMMS in a low resource setting.

While preparing the project, we realized that available international nomenclatures for biomedical equipment did not optimally match the concrete needs of developing countries. Although the ICMD classification showed larger gaps (e.g. for the identification of disability devices, medical furniture or energy supplies), fewer ambiguities were identified than with GMDN. The simplicity of the ICMD classification also better suited the available local coding competences. In order to achieve a better fit, further research will be needed to either generate a simplified sub-classification of GMDN or a more extensive version of ICMD. In any case, the free availability of such classification system seems to be an important factor for its usefulness in most of the sub-Saharan African countries.

Drawing up national standards for biomedical equipment and infrastructure that are adapted to the local health care system has proven to be a necessary and interesting exercise. Without such reference framework, solid biomedical heritage planning seems virtually impossible [2, 5]. However, due to the very difficult political and budgetary situation of Burundi during the complete study period, it has not been possible to adequately evaluate the usefulness of the developed framework, because no substantial biomedical investment decisions have been made at the level of the Ministry.

On the technical side, the use of a web-based system with a central server has generated very few difficulties in Burundi, despite frequent interruptions in internet connectivity due to power outages around the country. Admittedly, there is rarely a compelling need to have biomedical inventory and maintenance data available in real time, and in the event of system failures, data can either be entered quite easily at a later stage or recorded on an off-line computer which will then be synchronized afterward. Yet, periodic synchronization of off-line computers remained problematic in a few remote areas where no reliable mobile network coverage was available.

Since the health care system in the provinces of Muramvya and Kirundo had been supported for quite some time by the Belgian Development Cooperation, the studied health facilities can generally be counted among the better equipped public health care structures of Burundi. This partly explains why the *functional capacity ratio F* left less room for improvement from the start. It may be expected that the F value would rise more strongly in less fortunate areas, given the fact that some authors state that between 40 and 70% of complex medical equipment is lying idle in sub-Saharan African countries [4, 5]. The study data suggest that the improved *functional capacity ratio F* is caused by the intensified preventive maintenance activity. Further investigation also learned that the reason for that better F-score should in no case be sought in an increased number of decommissioned non-functional assets (which is theoretically possible and even desirable). On the contrary, decommissioning of assets continues to be a difficult administrative and cultural problem, even with an operational CMMS in place. The surrounding areas of many health facilities therefore remain littered with discarded defective equipment, which cannot be disposed of due to the lack of practical decommissioning procedures.

At the end of the study the MoH has been actively using the CMMS to determine its needs of infrastructure and medical equipment in consultation with its donors, in the context of the COVID-19 pandemic. In March 2020, after the first covid-19 cases in Burundi were identified, an overview of available CPAP ventilators, ICU beds and resuscitation equipment in the public health care facilities was immediately available and essential for the organization of the COVID-19 response.

The positive impact of action research in Kirundo and Muramvya led the Ministry of Health to include the roll-out of the software to the entire health system in its National Health Development Plan (2019–2023). However, the observed improvements come from a broader, successful combination of (1) completed maintenance teams with maintenance technician in 6 districts, (2) upgrade of technical platforms and workshops; (3) set-up of an operational frame with logistics for outreach activities to health centers; (4) use of a computerized maintenance management system (CMMS), (5) setup of a funding model.

Some of the donors have already committed to further expanding the CMMS to other parts of the country and a baseline inventory has already been entered into the system covering 17 of the 18 provinces for equipment and 11 provinces for infrastructure assets.

### Conclusion

This study provides strong evidence for the hypothesis that a sustainable implementation of a CMMS is feasible and useful in low resource settings, if the implementation approach (i) is done in a conducive technical environment with correct workshops and maintenance equipment, (ii) ensures the active cooperation of the central and decentralized administrative authorities, (iii) sufficiently long and repetitive training efforts are made, (iv) necessary hardware and internet connectivity is available and (v) adequate local technical support can be provided.

A well-functioning CMMS may have relevant impact on the functionality of the health facilities, and by extension on the resilience of the health system. It indirectly contributes to a greater availability and equity of qualitative medical care and to universal health coverage in general.

**Table 1:** CMMS implementation timeline

09/2016	CMMS functional & technical analysis
01/2017	First version of CMMS installed
04/2017	Going live of CMMS system
05/2017	GMDN nomenclature mapping
09/2017	CMMS features update and setup of on-site coaching program for CMMS users
01/2018	Production of a quick user manual for the CMMS
06/2018	New CMMS module for validation of inventories against national standards
10/2018	Recruitment of 2 CMMS coaches for on-site assistance
04/2019	CMMS transferred to MoH data center
09/2019	CMMS in the National Health Development Plan
01/2020	ICMD nomenclature mapping; new digital maintenance library added to CMMS
